# Spermatorrhœa in Gonorrhœa

**Published:** 1887-03

**Authors:** 


Spermatorrhœa in Gonorrhœa.—In studying the causation of spermatorrhœa, Fürbringer finds that a previous attack of gonorrhœa is the cause of spermatorrhœa in the majority of cases, as compared with the neurasthenic spermatorrhœa. He has been able to determine this fact for himself by a careful examination, during four and a-half years past, of the urine of patients under treatment for chronic gonorrhœa. In making his examinations, he has been careful to exclude cases in which the presence of a small quantity of semen could be attributed to natural coitus, or nocturnal emissions, or the practice of onanism, in the correct sense of the word, or of masturbation, as well as those cases in which straining at stool, or in any other way, has forced a little semen from the vesicles. Fürbringer has found in one hundred and forty cases of chronic gonorrhœa no less than twenty-five patients with what he calls "latent spermatorrhœa," that is, with the presence of a few spermatozoa of gonorrhœal in the urine, totally unsuspected in themselves. Fürbringer's investigations seem to indicate that in the spermatorrhœa origin, the discharge is not mixed with the secretion of the prostate, is devoid of the characteristic odor of the seminal fluid and contains no Böttscher's crystals, while all these are present in neurasthenic spermatorrhœa.—*Medical News.*

